# HIV-Exposed Uninfected Children: A Systematic Review on Psychological Well-Being and Association with School Performances in Africa

**DOI:** 10.3390/ijerph20032499

**Published:** 2023-01-31

**Authors:** Marina Mensi, Alain Ahishakiye, Katharine Journeay, Roberto Baiocco, Theresa Stichick Betancourt, Giacomo M. Paganotti

**Affiliations:** 1 Department of Developmental and Social Psychology, Sapienza University of Rome, 00185 Rome, Italy; 2 Capacity Building Team, Mental Health Centre, University of Rwanda, Kigali P.O. Box 4285, Rwanda; 3 Section of Epidemiology, Department of Public Health, Institut de Santé et de Développement, Cheikh Anta Diop University, Dakar 10700, Senegal; 4 Department of Social Medicine, Harvard University, Boston, MA 02115, USA; 5 Research Program on Children and Adversity, School of Social Work, Boston College, Boston, MA 02467, USA; 6 Botswana-University of Pennsylvania Partnership, P.O. Box 45498, Riverwalk, Gaborone, Botswana; 7 Division of Infectious Diseases, Perelman School of Medicine, University of Pennsylvania, Philadelphia, PA 19104, USA; 8 Department of Biomedical Sciences, University of Botswana, Private Bag UB 0022, Gaborone, Botswana

**Keywords:** depression, education, HIV-affected children, resilience, school performances, self-esteem

## Abstract

There is a growing number of children affected by HIV in Africa. Research on HIV-exposed uninfected children (HEU) is also growing. This systematic review focuses on the psychological well-being of HEU and its association with school intervention, outcomes, and enrollment in the African context, which is where the rate of HIV reaches its highest levels. Research on public databases was conducted according to PRISMA standards. Only studies on HEU primary school children in Africa, both quantitative and qualitative, were included. Out of 1510 papers retrieved, 50 met the inclusion criteria. These studies demonstrate that HEU children are more likely to perform worse in school compared to their counterparts who were not exposed to HIV and to show poorer concentration in the classroom. Children with parents suffering from AIDS are worried for them and have to take household responsibility, resulting in school dropouts, juvenile work, and risky behaviors. Few interventions have been conducted in the school environment with some of them being successful; therefore, future research should involve schools to create an inclusive environment where HEU children could enhance their potential and improve their psychological health.

## 1. Introduction

With increasing global access to Antiretroviral Therapy (ART), HIV has become a chronic illness. One of the consequences is a large growing number of HIV-exposed uninfected children (HEU) worldwide, especially in Africa, the world’s epicenter of HIV/AIDS. Moreover, as infected parents live longer, the main health and social outcomes of HIV have an even broader impact on families and communities. Young people may live with sick parents, face the death of one or both parents, or experience adversities such as the increased risk of school dropout, as well as taking on additional responsibilities at home. These challenges may also cause psychological problems. The present review aims to highlight the psychosocial health of HEU children and their families and to focus on psychological interventions conducted for HEU children at school and in other settings where children spend their time.

The vast majority of people living with HIV reside in low and middle-income countries (LIMC) and approximately 67% of them live in sub-Saharan Africa [1]. In Africa, the most affected countries are Eswatini, with 26.8% prevalence among adults [2], Lesotho, with 22.8% [3], South Africa, with 19.0% [4], and Botswana, with 20.7% [5]. West and Central Africa have a much lower rate of people living with HIV [1]. Nevertheless, Nigeria has almost 2 million people infected with HIV because of its exceptionally large population [6].

In 2019, there were 1.3 million HIV-positive pregnant women globally [7]. For many of them, pregnancy was the gateway for HIV diagnosis [7]. The risk of vertical transmission by an infected mother, in the absence of intervention, is estimated to be 15–30%, with an additional transmission risk of 10–20% when infants are breastfed [8].

Prevention of Mother-To-Child Transmission (PMTCT) interventions reduce the risk of transmission to less than 5% if strategies are pursued during pregnancy, labor, delivery, and breastfeeding [8]. Infants being born from HIV-infected mothers who underwent PMTCT intervention and who are virus-free are defined as HEU. In 2018, there were an estimated 14.8 million HEU children globally, 13.2 million of whom resided in sub-Saharan Africa [7].

In Africa, PMTCT interventions for HIV were first applied in South Africa in 1998–1999 in two midwife obstetric units, even though PMTCT was not yet officially implemented as a national policy [9]. Nowadays, PMTCT policies have been implemented in all African countries, with different successes and impacts. Therefore, there is a large, growing population of HIV-negative newborns being exposed in utero to the virus and to antiretroviral drugs used for prevention in the affected populations [10]. In general, the highest prevalence of these HIV-negative (but exposed in utero) newborns is in Southern Africa, where there is also the highest prevalence of maternal HIV [7]. 

Despite being virus-free, there is evidence that HEU children are at higher risk of mortality as well as for morbidity for common infectious diseases, slower early growth, developmental delays, and mental health problems when compared to their HIV-unexposed counterparts [7]. For example, a study carried out in the Democratic Republic of Congo showed that HEU children (at preschool age) demonstrated moderate to severe delays in mental, motor, and language development compared to HUU children [11] and several studies in various African countries have also evaluated neurodevelopmental outcomes of very young HEU children (aged between 12 and 24 months) [12,13] with contrasting results. However, outcomes of the studies support the view of program guidance for HIV-affected children that suggest family-oriented, home-based care [13]. Furthermore, a recent study from South Africa [14] showed that HEU children are at high risk of receptive and expressive language delay at the age of two. This study also highlighted the importance of long-term follow-ups in order to study the developmental outcomes of HEU children in sub-Saharan countries. ART exposure in utero among HEU infants has also been associated with growth faltering in a study from Botswana [15], as well as with neurological impairment in a study from South Africa [16]. It is essential to point out that a number of studies on childhood developmental status have demonstrated that several other factors might contribute to putting children at risk of poor development. These include low maternal schooling, violence against children, and, precisely, exposure to poor adult health and well-being [17]. 

It should therefore be highlighted that important contributing factors that undermine the mental health of HEU children are caused by parental illness or death, which often results in a significant reduction in child care. Many children under the age of 18 have lost one or both parents, which leads to consequences such as older children taking care of the youngest, children being in charge of their homes, and children dropping out of school [18]. Importantly, poverty and HIV seem to be correlated with child labor and early school leaving. The need for money in order to take care of their families’ impoverished situation can lead children to become involved in commercial activities with many negative consequences, such as child abuse and sex work [18]. Moreover, ill parents tend to become overprotected by their children, who therefore shift their role and responsibility from children to caregivers. The range of consequences is wide. Several studies indicate that HEU children are at risk of mental health problems, including depression, anxiety, social issues, low self-esteem, and stigma [19]. In addition, HEU children face many repercussions, such as economic insecurity, caregiver depression, and physical impairments [20,21,22]. HEU children often experience poor educational outcomes, with little chance to attend school. Alternatively, if enrolled, they may not attend regularly due to their home duties. Most of them are constantly worried about their sick parents at home; this causes concentration problems in the classroom, which undermine the student’s school success [23]. A systematic review at a global scale of HEU children’s school outcomes describes how the affected children drop out of school when a family member is ill or dead [24]. There are also occurrences of children not being enrolled in school at all, as caregivers give preference to only one child, deemed to be the smartest in the family, to attend school. In the same review, school outcomes are considered using different variables: school enrolment and attendance, age-appropriate grades, school performance (only for one study in China), school behavior, and goals achievement [24]. 

The growing number of HEU children in Africa, besides having a significant impact on HIV epidemiology, also has consequences on education. School is a place for socialization, where children learn by observation and identification, and acquire social–emotional skills, social norms, and behavior codes [25]. Furthermore, teachers may be important for vulnerable children to provide relief and discuss their burdens and worries. Effective schools have been commonly identified as being protective for high-risk children [18] and because schools are charged with educational goals, if children perform well in school, they may hope for a better future and job and decrease the possible chance to engage in risk behaviors [20]. 

The present review aims to organize papers on existing research about the connection and correspondence between the psychosocial health of HEU children and their families and how the school responds and intervenes. Research questions of interest are (a) What are the most common psychological problems identified among HEU children?; (b) How, if at all, have psychological problems among HEU children been linked to school functioning and other elements of well-being?; (c) What, if any, psychological interventions have been conducted for HEU children?; and (d) What, if any, interventions have been delivered in school and other settings where children spend their time?

## 2. Methods

### Literature Research

This systematic review follows the guidelines outlined by PRISMA 2009 checklist [26]. To identify papers on HEU children in Africa and related mental health and/or psychological problems in the school context, several databases were consulted: PubMed, PsycINFO, and Google Scholar. The search comprised: (1) a complete search of PubMed, PsycINFO, and Google Scholar databases; (2) an examination of the references from relevant papers; (3) a search of the articles of the authors in this field of research; and (4) efforts to contact authors working in the fields regarding published and unpublished studies (choices were based on the relevance and number of published studies as well as on authorship in highly relevant journals).

Inclusion criteria were (a) HIV-exposed uninfected children; (b) HIV-exposed uninfected children AND Africa; (c) HIV-exposed uninfected children AND Africa AND well-being; (d) HIV-exposed uninfected children AND Africa AND mental health AND resilience; (e) HIV-exposed uninfected children AND Africa AND risk and protective factors; (f) HIV-exposed uninfected children AND Africa AND developmental disabilities; (g) HIV-exposed uninfected children AND Africa AND developmental disabilities AND school outcomes OR performances; (h) HIV-exposed uninfected children AND Africa AND developmental disabilities AND school outcomes OR performances AND neuropsychology; and (i) HIV-exposed uninfected children AND Africa AND developmental disabilities AND school outcomes OR performances AND language delay. 

Studies located outside Africa and not mentioning school performances or any kind of connection with the criteria mentioned above were excluded. Moreover, to be incorporated, all studies had to be published in peer-reviewed journals from 2000 to 2021 (a few years after introducing the PMTCT), be written in English, and include children aged 6 to 18.

In addition, the NIH RePORTER database has been consulted for ongoing research projects. The keywords were HIV-exposed uninfected children AND cognition AND neurocognition AND mental health. Restrictions were age between 6 and 18 years, and be based on African countries.

**Figure 1:**
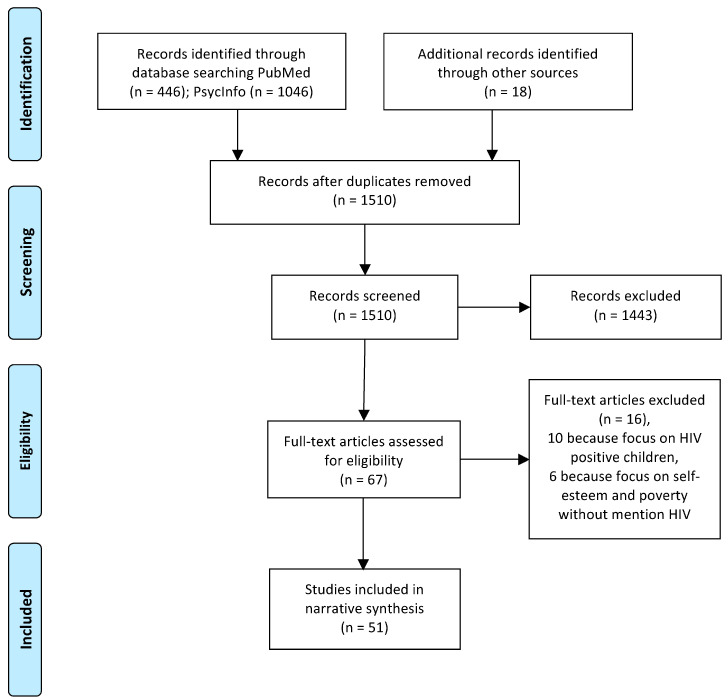
Systematic review following PRISMA standards.

Figure 1 shows the systematic review process following the PRISMA flow diagram. In total, we found on PubMed 446 papers about HEU children; 412 studies were excluded because they refer to medical research; 34 titles were eligible and fully read, of which 10 did not satisfy the inclusion criteria, resulting in 24 studies being included. A total of 1046 studies were found through PscycInfo, of which 1031 were excluded as considered irrelevant to the topic, 15 studies were valued for eligibility, and 9 were included. Two papers were eliminated because of duplication. An extra 17 studies were added after completing the bibliography by mining and checking the work of authors expert in the field. A final number of 50 studies were used for this review, in which both qualitative and quantitative studies were included. It is important to clarify that in this review terms and acronyms are used according to specific criteria: (i) HIV-negative children born from an HIV-positive mother are defined as HEU; (ii) children living in an HIV environment, either born from an HIV-positive mother or not, have been defined HIV-affected children, even though they are not HIV-infected by definition; (iii) AIDS orphans are also considered part of the HIV-affected group, even if papers do not clearly specify whether they are HEU or HIV-infected; (iv) the category of HIV-unexposed uninfected children (HUU) corresponds to a group of HIV-negative children who were born from HIV-negative mothers and were therefore never exposed to HIV; and (v) sometimes, HEU children may fall under the category of HIV-affected children.

## 3. Results

A summary of all the studies is presented in Table S1. Fifty (50) papers were retrieved for the purpose of this review. All studies were conducted in sub-Saharan African countries and particularly: Botswana, Eswatini (ex-Swaziland), Kenya, Malawi, Namibia, Rwanda, South Africa, Uganda, Zambia, and Zimbabwe. Only one study was from North Africa, namely, Egypt.

This review focuses on the psychological health and well-being of school-aged children related to school outcomes and teachers’ support. Nevertheless, to achieve a comprehensive picture, we also addressed psychological health and well-being itself. 

Results are organized into the three main factors: (1) individual factors; (2) family factors; and (3) social factors and school environment. The last section describes interventions aiming to improve the psychological well-being of HEU children as well as affected families. 

### 3.1. Individual Factors

The psychological health of HIV-affected children is a topic that researchers frequently address, and many studies suggest that the affected children are at high risk of psychological distress and behavioral problems [27].

### 3.2. Self-Esteem and Resilience

Self-esteem is considered a part of resilience [28]. As a matter of fact, children with high self-esteem can better cope in stressful environments while children with poor self-esteem are at greater risk of depression and anxiety [28]. Furthermore, community violence and trauma are associated with lower self-esteem [29]. A high score of self-esteem corresponds to a high level of Perceived Social Support (PSS). Regular school attendance and educational opportunities are predictors of a high score of PSS, which refers to school as an effective setting to generate a support network [30]. 

Mental health resilience may be associated with multiple factors across children, family, and community. Child physical health, caregiving support, food security, peer relationship quality, and lower exposure to community violence and bullying or stigma seem to be strong predictors of resilience [31,32]. Moreover, a study that was carried out in Egypt suggests that children and adolescents of HIV-positive parents had a significantly lower health-related quality of life than children of healthy parents [33]. This may be attributed to the effects of parental HIV on the family and the economic and social issues that follow [33]. 

### 3.3. Depression

Several studies include depression scales in their research, and they usually report depression correlated to different circumstances. Multiple factors may lead to high levels of depression in HIV-affected children. Studies report instances of family behavior, such as domestic violence, physical abuse, and daily harsh punishment, as the main responsible factors for high-level depression [20,28,34]. Domestic violence between adults seems to be a predictor of depressive symptoms aggravation [29] and it appears to be consistently present in HIV-affected families, due to the high level of stressors. An example of family stressors may be the correlation between caregivers’ physical health and their caregiving capacities: children with a high level of depression and anxiety are those whose caregivers reported mental and physical impairments [22,35].

One of the first quantitative studies on psychological health in South Africa [36] demonstrated that HIV orphans had a higher level of depression, post-traumatic stress, and suicidal intention when compared to orphans from other causes and non-orphans. However, there were no significant differences in anxiety levels among the three groups. Another study from South Africa [37] about the effects of stigma on mental health, showed that AIDS-related stigma strongly conveyed the association between AIDS orphanhood and depression. Poverty, measured through school access, food security, household employment, and household receipt of welfare grants, was another factor that conveyed the association between AIDS orphanhood and depression [19]. Further study that was carried out in a deprived, violent, urban South African setting, demonstrated that the interaction among poverty, AIDS-related stigma, and bullying were predictors of depression and other psychological problems in HIV orphans [38]. Data confirmed that food insecurity and related stigma increased the percentage of children with mental health disorders from 19% to 83% [38]. A qualitative study from Rwanda [39] presented an analysis of mental health among HIV-affected children. The study employed open-ended interviews with adults (clinicians and social workers) and children. Ninety (90) % of the clinicians reported persistent irritability and anger in HIV-affected children and described children as always annoyed, grouchy, and not appreciative of anything. Additionally, they were reported to talk rudely and to quarrel. The study also showed that affected children often performed poorly at school and were isolated from other children [39]. 

A cluster randomized controlled trial study from Uganda with case and control groups showed a significant reduction in depression when using an economic intervention [20]. Moreover, the authors found that depression symptoms were possibly related to different variables: old age of caregiver, female gender of caregiver, and poor physical health [20]. A study from South Africa tried to reduce the level of depression by using home visiting by specialized persons, but results did not show any change from the baseline to the follow-up, and depression continuously persisted among HIV-affected households [40]. In a gender study based in South Africa and Malawi, depression scores and suicidal ideation did not differ between male and female children [41]. Another study from South Africa showed a high level of depression in HIV-affected children, especially boys between 11 and 25 years old, and a possible association with poor educational outcomes [42].

### 3.4. Gender Differences in School Outcomes

Few studies described the difference in school outcomes between male and female HIV-affected children. Some of them underlined that boys are at higher risk of having poor outcomes when compared to girls. In the results of the aforementioned study by Orkin et al. [42], male students were directly associated with concentration issues and consequent difficulties in grade progression, both in HIV-affected children and orphans from HIV. Consistently, in another study, female HIV orphans showed significantly lower mental health problems than boys [43]. The same gender differences in learning outcomes were confirmed in the study from South Africa and Malawi [41], where it appeared that boys struggled more in school and were slower learners than girls. The study also underlined the importance of involving boys in educational activities in order to reduce the possibility of engaging in risk behaviors [41]. Girls seemed to be more likely to improve their condition when they could benefit from some facilities, as appeared in a study carried out on students who received a grant [44]. As a matter of fact, receiving money and care was associated with lower educational risks. On the other hand, grants had an inferior impact on boys’ improvement, concerning both their learning speed and their overall school outcomes [44]. Another study from Uganda examined the gender differences in children regarding the effects of parental loss due to HIV/AIDS [45]. Findings showed that after their mothers’ deaths, girls were more likely to take care of younger children and the surviving parent in the house than boys. However, they also reported that girls consequently had less food and money as their duties prevented them from having easy access to goods of first necessity. Conversely, girls became more scared after the death of their father as compared to boys, who were more determined to take matters into their own hands after the death of their father. However, despite receiving increased duties, boys did not receive the same workload as girls [45].

### 3.5. Cognitive Abilities

A study on working memory was conducted with three groups of children, HIV-infected, HEU, and HUU, in South Africa [46]. It demonstrated that HIV-infected and HEU children manifested a similar low pattern on verbal storage and verbal processing tasks, but while HIV-infected children had a more complex physical situation, HEU children’s situation may lead to language difficulty. For visuospatial working memory, the score was the same as for the HUU children [46].

Cognitive abilities were also measured with draw-a-person tasks in a cash grant intervention study from South Africa [44]. Results showed that cognitive abilities were associated with being in the correct grade at school and attending school regularly. Another study from Uganda determined how HIV infection predicts a deficit in cognitive executive functions among school-aged children exposed or not exposed to HIV [47]. Results showed that HEU children had significantly greater executive function deficits than HUU children, suggesting that perinatal exposure to HIV, even without infection, negatively affects executive functions [47]. In addition, a different study from Uganda examined the quality of life of HIV-positive, HEU, and HUU children [48]. Tests included a Cognitive Function Test, the results of which showed that only HIV-positive children obtained the lowest scores, whereas HEU and HUU children are on the same level on the entire Quality of Life subtest [48].

The NIH RePORT website describes three ongoing studies on the cognitive and behavioral function of primary school-aged HEU children, both alone and compared with HIV-positive or HUU in Africa. Boivin JM (Michigan State University, East Lansing, MI, USA) explores whether Brain Powered Games cognitive assessment data, gathered while HIV-affected children (aged 5 to 12 years in Uganda and Malawi) play, can be analyzed to understand how HIV risk factors affect the developments of a child’s brain [49]. Another ongoing study in Botswana carried out by Lowenthal ED (Children Hospital of Philadelphia, Philadelphia, PA, USA) explores whether HEU and HIV-infected children (primary school-aged) are more likely than their peers to have neurocognitive deficits. This will be determined by the implementation and validation of a computerized neurocognitive testing battery to identify neurocognitive problems [50].

Finally, Robb ML (Henry M. Jackson Foundation, Bethesda, MD, USA) aims to explore determinants of resilience (defined as “competence during adolescence in cognitive, emotional and behavioral functioning in the face of adverse circumstances”) in adolescents living with or affected by HIV in a comparative study also including a South Africa cohort [51].

### 3.6. Family Factors

#### Family and Parental Support

It has been shown that HIV physical illness affects parents in many ways: their interest in child care decreases, parental absence increases, and children are neglected. HIV-affected families are at elevated risk of adversities, thus resulting in increased child behavioral problems [35,52]. Depressed mothers of children affected by HIV/AIDS in Uganda were more likely to abuse and neglect their children, as underlined by Kagotho et al. [53] where it has been reported that women were more at risk to show serious depressive symptoms when compared to men. Thirty-five percent of the respondents had household savings, and those without any savings reported depression symptoms [53]. A large survey on HIV/AIDS’s impact on parenting behavior in South Africa confirms the statement above and highlights that the AIDS condition is associated with less capacity to take care of children [54]. The study reported that poverty is often present and relevant in HIV-affected families due to unemployment and medical and funeral expenses. These factors increase the caregiver’s depression and limit their capacity to engage positively with their children [54].

Another survey, conducted in Uganda on children orphaned by HIV/AIDS aged 10 to 16 years, explored the impact of parental loss on their well-being. Participants reported worsening school grades and a decline in school attendance. After losing their mother, there was an impact on their family’s basic needs, while after the death of their father, the majority of children reported feelings as being lonely, isolated, worried, angry, and scared. Nevertheless, participants also reported feelings of comfort and relief following their parental loss (because they ceased to worry about their HIV-sick parents) and determination to do well in many fields of life [45].

In South Africa, a study by Sherr et al. [34] examined differences in double or single parental death. Results showed that double orphans were significantly more likely to be kept out of school to help with family chores, while children with a single parental death and children with no death were kept in school.

Two studies, by Ashry et al. [33] and Casale et al. [55], described that caregivers’ higher education was associated with fewer adolescent emotional and behavioral problems. Moreover, if caregivers received more support, adolescents’ problems could be further reduced. The findings in these studies reinforce the importance of promoting caregivers’ social support to improve parenting and reduce the risk of adolescents’ behavioral and emotional problems [33,55].

Although the aforementioned studies focus mainly on the psychological issues of children living in an HIV environment, a study from Uganda [56] underlined the role of children as important supportive figures for HIV-positive parents. In fact, Nalugya et al. [56] showed that the role of children involved in the care of HIV/AIDS parents seemed to influence their sense of commitment, maturity, and self-esteem.

Another study from Uganda [57] examined caregivers’ mental health, both on HIV-positive and negative caregivers, and their impact on child well-being. Results showed that there was no direct effect between HIV-positive caregivers and children’s well-being. Instead, data highlighted how depression and anxiety (with or without HIV) compromised the quality of caregiving in many situations and therefore created problems on the five well-being indicators of children: distress, hopelessness, positive future orientation, self-esteem, and quality of life, in children aged between 6 and 18 years old.

### 3.7. Social Factors and School Environment

#### Risk and Protective Factors

A study from South Africa with 100 children aimed to explore possible differences in children’s psychosocial well-being between two groups, one living in a homestead and one living in a shelter/hostel [58]. No differences were found between the living arrangements of children (i.e., hostel vs. homesteads) despite hostel children coming from the most vulnerable situations. 

However, as far as social support (defined as access to trusted adults) is concerned, those with social support had significantly higher rates of positive well-being and emotional attachment. In general, children living in homesteads presented a higher frequency of negative experiences such as going to bed hungry, suicidal ideation, and feelings that they did not live in a safe place [58]. Given these differences and therefore the idea that living arrangements importantly impacted the development of children, it was expected that access to trusted caregivers would be a major protective factor. 

In 2004, UNICEF issued a document where the characteristics of protective schools were described as follows: (i) quality learners, healthy and well-nourished children; (ii) quality content, well organized, and proper curricula and materials; (iii) quality teaching-learning processes, such as technology child-centered and skill-based approaches; (iv) quality learning environment, which adopts protective policies and practices as well as provides appropriate facilities and services; and (v) quality outcomes and evaluation, which is the achievement of relevant knowledge, attitude, and skills on the children’s side, as well as classroom assessments, which have to be adapted to national levels [59]. In the HIV context, protective factors are associated with school enrolment and attendance, while risk factors are mostly related to poverty. Two papers considered both risks and protective factors within the school context [60,61]. Protective factors were mostly associated with the feeling of happiness at school, caregiving support, and community support. Scorza et al. [61] considered resilience, patience, perseverance, self-esteem, tenacity, adaptation, and spirituality as important factors to accept adverse situations. Risk factors were commonly related to poverty, unhappiness at school, assisting sick caregivers, emotional distress/suicidal ideation, crime and substance abuse, adolescent health, and several traumatic events [61]. AIDS orphans exhibited a higher level of delinquency and conduct problems when compared with non-AIDS orphans [38]. Furthermore, AIDS orphans showed a higher tendency to school dropout when compared to their non-AIDS orphan counterparts. Additionally, they were less likely to live in a household, to receive state grants, or to have access to welfare support [38]. 

Although the school might seem to be the place where most of the children’s needs should be met, it is also where children may experience troubles, such as stigma, bullying, and poor interactions with peers. A study from Namibia and Swaziland [18] reported that schools generally did not meet HEU children’s needs and most of them dropped out of school due to teachers’ violent behavior and ostracism from their teachers and peers. In addition, social issues such as bullying, discrimination, and stigma for HIV families were very frequent, and teachers also confirmed that those were among the main causes for dropping out of school [18]

Studies from Kenya and Zimbabwe [23,62] showed that the lack of supportive adults, household responsibilities, and child abuse and neglect impacted school attendance in HIV-affected children. In particular, the qualitative study from Kenya [23] collected teachers’ interviews, which mostly explained that pupils had to provide medicine and food for their HIV-sick parents every day. Moreover, if caregivers were bedridden, they also had to take care of their basic needs, such as hygiene. Taking care of their sick parents left children exhausted, both mentally and physically, and most of them did not receive enough food, which made them feel lethargic, tired, and anxious: concentrating at school therefore became a problem [23]. It should also be considered that when children were at school, they continuously worried about their home situations and lived in fear of finding their parents dead [62]. Another study, from Botswana [63], showed that HIV-affected children were usually very happy, but those who were unhappy reported they did not like going to school because of bullying, stigma, teasing, and poor interaction with other children and teachers.

In a qualitative study [62], students from a school district in Zimbabwe were asked to write a story about a child in their school who was affected by HIV by describing challenges and difficulties in their everyday life. Results showed how children conceived an HIV environment: visibly poor houses, lack of food, etc. It also demonstrated how children coped in the school context by analyzing their teachers’ support or negative responses, their peers’ support, and their social exclusion [62]. Overall, the stories described the emotional impact of HIV on HEU children and the limited support provided by schools. Specifically, the difficulty in establishing relationships and support networks, and the fear of stigma from peers and teachers even if they recognized their suffering.

Most people affected by HIV are school-aged HEU children. School has a pivotal role in their growth because, often, it is the only place where they can have their needs addressed [63]. Many children use school as a distraction from life tragedies. They associate their home with negative emotions and their school with positive emotions, where they can play freely without any family burden [62]. School is identified as the institutional setting where children are regularly engaged the most and where teachers should be the main source of support [24,62,63,64]. In an interview with HIV-affected students from Botswana, children expressed their desire to improve their school grades, receive better scholastic material, obtain more support from their teacher, have better school meals, eliminate bullying and teasing, receive more understanding/love from their teachers, and benefit from better teaching approaches [63].

### 3.8. Educational Outcomes

Research on educational outcomes considers a range of outcomes, including school dropout, failed enrolments, attendance of grades addressed to younger students, poor behavior at school, general school performance, and grade attainment [24,65].

In a study from Zambia with two cohorts of primary school-aged children [66], authors examined the physical growth and the general health outcomes of 111 HEU and 279 HUU children together with school reports. The study showed that there were no differences in the English score between HEU and HUU students, while HEU students showed a lower score in Mathematics compared to HUU students. 

A study from Botswana on HIV orphans (it is not specified whether the children were HIV-positive or negative) showed that most children had access to school and they liked it because they thought they had good teachers and good quality education [67]. Another (pilot) study from Botswana showed improvement in child resilience thanks to a program created by an NGO in order to promote resilience among vulnerable children. This program consisted of a camp where traditional local approaches were integrated with typical Western treatments, such as group psychotherapy [68]. Moreover, Adler’s study [25] showed the importance of having at least one best friend in another context. This would protect children and adolescents from being bullied. In fact, some children did not like school because of corporal punishment and bullying from other students [67]. These issues negatively affected school grades, which, however, met the letter C criteria (reference to scores criteria A, B, C, D, and E, being A outstanding, D unsatisfactory, and E lacking effort or interest) [67]. 

A paper from South Africa examined the greatest difficulties encountered by adolescents [52]. Problems included concentration at school, hunger at school, missing lessons, and dropping out of school. Quantitative analysis provided evidence that adolescents living in a household with HIV/AIDS sick relatives or caregivers were more likely to experience those problems and tended to be more vulnerable to educational challenges compared to those living in a healthy environment [52]. 

In line with the aforementioned study, it seems that the main impairing factor for HIV-affected school-age children in Zimbabwe was socioeconomic, and further findings showed that most of the vulnerable children were experiencing financial constraints [65]. The authors suggested that investing in cash grants could be a good solution for HEU children to promote their well-being and school progress [65]. 

### 3.9. Interventions

A quasi-experimental, cross-sectional, post-intervention survey carried out in South Africa, evaluated the use of an art program to increase self-esteem and self-efficacy in HEU children [28]. Children attending the program significantly increased their self-efficacy, but did not show any improvement in self-esteem, depression, and emotional or behavioral problems [28].

Another study from South Africa [64] introduced an intervention to build positive relationships between parents and children. The aims were to promote the well-being of HIV-positive mothers and to improve their interactions with their children. Many activities were created for them to bond. In fact, traditionally, African parents do not play much with their children. A three-month follow-up showed that there was a growth in parent-child communication. Parents felt more open to understanding their children without using any physical punishment and they were able to help their children with their homework. 

Differently, a longitudinal, quasi-experimental design from South Africa evaluated the impact of two home-visiting program models on the psychological health of HIV-affected children and their caregivers over two years [40]. Models were made by a group of paraprofessionals and a group of volunteers. After the two years follow-up, both models did not show any improvement in psychological health, which continued to persist and even worsen [40]. The intervention highlighted the challenges of HIV-affected families and the need for solid psychological support.

Betancourt et al. [21] developed the Family Strengthening Intervention (FSI) in Rwanda. At first, Betancourt’s FSI study was carried out to assess the acceptability and feasibility of the intervention in order to reduce mental health problems and to boost resilience among children living in households affected by their caregiver’s HIV in Rwanda. The intervention was adapted to the Rwandan context, which concerned the relevant mental health problems of the participants, which derived from their local and cultural situation. Therefore, it included psychoeducation on genocide-related trauma and it focused on overcoming past experiences in order to benefit from them and build improved present resilience. The FSI had four key components: building parenting skills and improving family communication; developing a family narrative, providing psychoeducation on HIV transmission, prevention, and normative responses; strengthening problem-solving skills; and social support. The intervention was conducted by counselors who first met the members of the family separately and, at a later time, altogether [21]. Post-intervention and six-month follow-up results indicated that caregivers presented improvements in self-esteem, perseverance, depression, anxiety, and irritability. Both children and caregivers also reported a reduction in harsh punishment after the 6 months follow-up [21]. Economic intervention with scholarships has been implemented in order to enhance children’s performances at school and consequently reduce depressive symptoms in HIV-positive caregivers. The results showed great improvements in Uganda [20]. Three other studies examined the implementation of scholarships to enhance school outcomes. The first study, still from Uganda, promoted an intervention based on monetary savings [69]. Results showed positive effects on academic performances and indicated that children who received economic support had higher possibilities of achieving their educational aims [69]. The other two studies, based both in South Africa and in Malawi, explored the effect of scholarships influence on HEU children’s educational risks and cognitive development outcomes. Children in households receiving grants showed higher regular attendance rates at school with a low number of absences and fewer chances to engage in risk behaviors [44]. Findings on cognitive outcomes showed that cash grants have potential effects on cognitive abilities as well. Furthermore, the authors evidenced that a combination of good parenting and a grant was a strong predictor of a higher score on cognitive performance [70].

## 4. Discussion

This paper aimed to explore and systematize the psychosocial well-being of HIV-affected and HEU children and its association with school outcomes. In particular, this review examines the dynamic process of individual, family, and social factors, and the interventions that shape school performances and educational outcomes.

Concerning the research questions proposed in the Introduction section, a summary is provided, based on evidence from the analyzed literature, as follows: (a) *What are the most common psychological problems identified among HEU children?* The most common psychological problems identified among HEU children are lower self-esteem and resilience, depression, and decreased cognitive abilities; (b) *How, if at all, have psychological problems among HEU children been linked to school functioning and other elements of well-being?* It has been shown that children’s depression and resilience are causally related to their caregivers’ or parents’ behavior in HIV-affected families. The more parents are mentally or physically ill, the more children become vulnerable to anxiety, depression, and high-risk behaviors [31,32]. This may lead to poor school performance, concentration issues, and general school failures [23,42,52]; (c) *What, if any, psychological interventions have been conducted for HEU children?* Family intervention seems to be the key to enhancing children’s psychosocial well-being in HIV-affected families [20,40]. In particular, when delivered by counselors [20], it has improved the relationship between parents or caregivers and their children; and (d) *What, if any, interventions have been delivered in school and other settings where children spend their time?* Few interventions have been delivered in schools. Altogether, it has been shown that the more children establish a positive relationship with teachers, the more resilient they are, and the more their school performance increases [25]. In fact, teachers may be a focal point for offering support and creating an inclusive environment among classmates and peers. As a result, students’ academic potential, self-efficacy, and self-esteem may become more strongly associated with academic outcomes [71]. 

Other important evidence of successful interventions is that concerning cash grants or saving accounts for families and children [44,69,70]. Among them, the most successful seemed to be those reinforcing good parenting (increasing family quality time between child and parent) in combination with cash grants [70]. Cash grants given to parents help the parents to build self-confidence and allow them to properly feed their children. Lastly, Sseswamala et al. [69] highlighted the importance of parents having the opportunity to use money to send their children to school without affecting their extremely limited resources.

Child gender was also relevant, particularly in given outcomes and responsibilities. In HIV-affected families, girls often take the entire house burden, which results in increasing school absences and eventually in school dropout, but thanks to the right financial support, they showed improvements in school outcomes compared to boys [41,42]. Boys were less likely to be engaged in household activities, but they were more at risk to leave school in order to start working, especially if their parents were sick and unable to work. Overall, boys seemed to show more school concentration and performance difficulties even when cash grants were implemented [42].

Several studies highlighted the dual role of school: as a risk and as a protective factor. In fact, besides their family and their socio-economic context, the school environment may be considered the largest source of risk factors, such as bullying, stigma, and lack of teachers’ support, as in the cases of Botswana [63,67] and Zimbabwe [62].

Furthermore, few studies explored cognitive abilities in primary school children [46,70], but more investigations need to be conducted in order to understand if poor school outcomes are caused by biological factors (such as the exposure to the virus and HAART in utero) or because of social circumstances. Exploring the cognitive abilities of school-aged children will be important, especially in the African context.

Alternatively, schools may also play a role as a protective factor, providing relief to children [60,62], and especially for those with family burdens and problems [60,61]. In the absence of easily available psychological support, schools are the only places where children can address their needs [63]. In fact, teachers may represent a focal point for offering support and creating an inclusive environment among classmates and peers. As a result, students’ academic potential, self-efficacy, and self-esteem may affect academic outcomes in a more significant way [71]: it has been shown that the more children establish a positive relationship with teachers, the more resilient they are and the more their school performance improves [25].

Furthermore, several studies evidenced how HEU children at the age of 24 months may exhibit developmental delays, especially in language-related areas [14]. Considering that language impairment is the core element of learning disabilities and usually does not disappear over time [72], it is essential to intervene and train teachers to cope with these problems.

We must remember that good education can lead to a chance to have a good job in the future, can reduce the menace of engaging in risky behaviors, and reduce the prospect of becoming HIV-positive [41,73]. 

Due to its characteristics and themes, the review shows some limitations. The number of papers is restricted, and the topic still needs further investigation. Studies controlling for cognitive abilities are primarily carried out outside of African countries and when they are based in Africa, the sample age is focused on infants. Similarly, the topic of psychological well-being itself is already present in many studies, but not yet related to school performances and outcomes. 

## 5. Conclusions

Overall, it is evident that HEU children experience several psychological, family, and social factors that seem to cause a number of problems with school performance and outcomes. Parents, caregivers, and teachers should cooperate to invest in good education. A greater focus on the school environment is needed in order to create a safe place for HEU children, where they can feel understood by teachers and peers. Providing the opportunity for HIV-affected children (including HEU) to meet their educational needs is extremely relevant, and different programs should be implemented to address their educational needs.

An inclusive environment should be created to assist children with schoolwork and to support them whenever they have difficulties. It is essential to address the needs of HEU children within the school environment and to create a welcoming space where they can feel accepted by teachers and peers. 

Similarly, there is a need to implement evidence-based school programs to guarantee robust learning for HIV-affected children and increase their chances to access to success in the future. 

These programs are increasingly urgent as the SARS-CoV-2 pandemic disrupts education and healthcare systems both in Africa and worldwide.

## Data Availability

Not applicable.

## Supplementary Materials

The following supporting information can be downloaded at https://www.mdpi.com/article/10.3390/ijerph20032499/s1, Table S1 summarizes the 50 studies analyzed in the systematic review.
