# Supplementary material for: HIV-Exposed Uninfected Children: A Systematic Review on Psychological Well-Being and Association with School Performances in Africa
Source: Int J Environ Res Public Health. 2023 Jan 31;20(3):2499. doi: 10.3390/ijerph20032499 (PMC9916290; doi:10.3390/ijerph20032499)
Supplement: Supplementary file 1 [file ijerph-20-02499-s001.zip › ijerph-1910428-supplementary.pdf]

| N | Author(s) | Country | Sample (N) | Age (years) | Domain assessed | Measurements | Type of study |
|---|---|---|---|---|---|---|---|
| 1 | Cluver et al., 2007 [36] | South Africa | 1025 children (425 orphaned by HIV/AIDS, 241 orphaned by non-HIV/AIDS causes, 81 orphaned by unknown causes, 278 not orphaned) | 10 – 19 | Individual | CDI; R-CMAS; SDQ using the 5-items peer problems subscale; Amaya-Jackson's Child PTSD Checklist; SDQ using the 5-items conduct problems subscale; CBCL YSR using the 11-items delinquent subscale | Quantitative study, interviews |
| 2 | Betancourt et al., 2011 [39] | Rwanda | 43 children (HIV/AIDS-affected) and 31 adults | 10 – 17 | Individual | Free-Listing interview: "What are the problems of HIV/AIDS-affected children in this community?" Key Informant Interviews: "Tell me more about the problem of *agahinda kenshi* (persistent sorrow) among HIV/AIDS-affected children in this community"; "How does a child with *agahinda kenshi* feel?"; "How does a child with *agahinda kenshi* behave?"; "How does a child with *agahinda kenshi* think about | Qualitative study |

| | | | | | | | |
|---|---|---|---|---|---|---|---|
| | | | | | | themselves or others?” | |
| 3 | Han et al., 2013 [20] | Uganda | 297 children (orphaned by HIV/AIDS) | 12 – 14 | Individual | BHS; CDI | Two-arm cluster randomized controlled trial |
| 4 | Sipsma et al., 2013 [35] | South Africa | Children (HEU) and 509 mothers (395 HIV-positive, 114 HIV-negative) | 6 – 10 | Individual | VABS; CBCL; CDI; RCMAS; BOEQI YV | |
| 5 | Betancourt et al., 2014 [27] | Rwanda | 683 children (218 HIV-positive, 228 HIV-affected, 237 HIV-unaffected) | 10 – 17 | Individual | WHODAS-Child; CESDC; YSR; anxiety/internalizing scale; Parental Acceptance and Rejection Questionnaire using 16-items; United Nations Children’s Fund’s Multiple Indicator Cluster Survey using 12-items; Post-War Adversities Index; SAFE child protection checklist using 17-items; HIV-related stigma reported on a 4-point Likert scale of *never*, *sometimes*, or *often/a lot*; HSCL | Case-control study |
| 6 | Govender et al., 2014 [22] | South Africa | 623 children (226 orphaned by HIV/AIDS, 397 not orphaned) | 9 – 15 | Individual | YSR; SACAS | Cross-sectional study |
| 7 | Barenbau et al. 2016 | South Africa | 100 children (vulnerable or | 12 – 22 | Individual | PAS-A; RADS-2; R-CMAS | The study investigated the |

| | | | | | | | |
|---|---|---|---|---|---|---|---|
| | [58] | | orphaned by HIV/AIDS) | | | | role of social support and attachment on positive well-being among orphaned and vulnerable children due to HIV/AIDS |
| 8 | Ezeamama et al., 2016 [47] | Uganda | 166 children (58 HIV-positive, 55 HEU 53 HUU) | 6 – 18 | Individual | BRIEF questionnaire | Comparative study |
| 9 | Milligan et al., 2017 [46] | South Africa | 273 children (95 HIV-positive, 86 HEU, 92 HUU) | Mean age: 7.42, 7.36, 7.05, respectively | Individual | AWMA; ELP | Case control study |
| 10 | Nkwata et al., 2017 [48] | Uganda | 168 children (58 HIV-positive, 54 HEU, 56 HUU) | 3 – 18* | Individual | PedsQL; medical history; physical examination, and laboratory evaluations | Retrospective cohort study |
| 11 | Lowenthal, et al., 2018 [50] | Botswana | 650 children (200 HIV-positive, 200 HEU, 250 HUU) | 7 – 18 | Individual | PennCNB; PSC | Case-control study |
| 12 | Robb et al., 2018 [51] | South Africa and United States of America | Three cohorts: HIV-positive, HEU, HUU | Adolescents | Individual | Higher cognition; executive, emotional and behavioural functioning; identify the determinants of resilience | Case-control study |
| 13 | Boivin, et al., 2019 [49] | Malawi and Uganda | Three cohorts: HIV-positive, HUU, HEU | 12 | Individual | BPG; CCRT | Case-control study |
| 14 | Cluver et al., 2012 | South Africa | 599 children | 10 – 20 | Social | Questionnaire: | Mixed qualitative |

| | | | | | | | |
|---|---|---|---|---|---|---|---|
| | [52] | | (212 with HIV/AIDS condition at home, 140 with other- conditions at home, 247 in healthy homes) | | | educational outcomes when controlling for sociodemographic s | and quantitative study |
| 15 | Campbell et al., 2014 [62] | Zimbabwe | 128 children | 10 – 14 | Social | Children story about HIV in the school context | Qualitative study draws and write story about HIV |
| 16 | Nabunya et al., 2014 [45] | Uganda | 1410 children (orphaned by HIV/AIDS) | Mean age: 12.7 | Social | Questions on the effects of parental loss on children's feelings: *1*) How has the loss of your father affected the way you feel about life? and *2*) How has the loss of your mother affected the way you feel about life? | Quantitative study; survey |
| 17 | Watkins et al., 2014 [60] | South Africa | 243 children (orphaned by HIV/AIDS) | 13 – 22 | Social | Open ended questions to elicit individual perspective on education; risk and protective factors | Qualitative study |
| 18 | Collishaw et al., 2015 [31] | South Africa | 944 children (425 orphaned by HIV/AIDS, 241 orphaned by non-HIV/AIDS causes, 278 not orphaned) | 10 – 19 | Social | CDI; RCMAS; CPTSDC; SDQ; CBCL | Longitudinal study |
| 19 | Skovdal, 2016 [23] | Kenya | 47 children (vulnerable children and | 10 – 20 | Social | Interview: school experiences of HIV affected children | Qualitative study |

| | | | | | | | |
|---|---|---|---|---|---|---|---|
| | | | orphaned by HIV/AIDS) and 18 teachers | | | | |
| 20 | Visser et al., 2012 [64] | South Africa | 45 children/ HIV-positive mothers dyads | 6 – 10 and 25 – 42 | Individual, and family | N/A | Qualitative study |
| 21 | Orkin et al., 2013 [42] | South Africa | 723 children (269 orphaned by HIV/AIDS, 103 with HIV/AIDS condition at home, 351 not orphaned) | 11 – 25 | Individual, and family | Educational outcome; poverty; CDI; RCMA; CPTSD | Quantitative study |
| 22 | Betancourt et al., 2014 [21] | Rwanda | 39 children and 28 caregivers | 7 – 17 and 30 – 70 | Individual and family | Family connectedness; good parenting; CD-RIS; pro-social behaviour; MICS; CESDC; ISSB; YSR; IRQ; YCPS-RS; WHODAS-Child | Pre-post design, including 6-month follow-up; qualitative and quantitative study; Intervention |
| 23 | Sherr et al., 2014 [34] | South Africa | 381 children (24 double orphans because of HIV/AIDS, 70 single orphans because of HIV/AIDS, 287 not orphans) | 8 – 19 | Individual and family | RSE; SEQ-C*;* CDI; SDQ; Adapted Community Maltreatment, Exploitation, Stigma & Discrimination Scale; P-C CTS; Externalizing and Risk Behavior Scale | Interview; quantitative study |
| 24 | Casale et al., 2015 [55] | South Africa | 2477 children/ caregivers dyads | 10 – 17 and >18 | Individual and family | MOS-SSS; APQ – short form; SF-12; SDQ | Cross-sectional household survey |
| 25 | Skeen et al., 2016 [29] | Malawi and South Africa | 588 children (HIV-affected) | 4 – 13* | Individual and family | CDI; TSCC; RSE; SDQ; SAHA P-C | Cross-sectional study |

| | | | | | | | |
|---|---|---|---|---|---|---|---|
| | | | | | | CTS; Child Abuse and Neglected screening tools | |
| `26 | Nalugya et al., 2018 [56] | Uganda | 38 HIV-positive parents | 18 – 61 | Individual and family | First interview (unstructured): life and illness history; second interview (semi structured): life history and HIV self-management; role of children with their parents | Qualitative study |
| 27 | Cluver et al., 2008 [37] | South Africa | 944 children (425 orphaned by HIV/AIDS , 241 by non-HIV/AIDS causes, and 278 not orphaned) | 10 – 19 | Individual and social | CDI; CMAS-R; SDQ; PTSD; CBC; Social and Health Assessment Peer Victimization Scale; self -report of past-year experience of witnessing or having been a victim of the study area's four most common community crimes; Berger Stigma Scale for HIV-positive youth–Revised | Quantitative study; interview |
| 28 | Cluver et al., 2009 [19] | South Africa | 944 children (425 orphaned by HIV/AIDS, 241 orphaned by non-HIV/AIDS causes, 278 not orphaned) | 10 – 19 | Individual and social | CDI; CMAS; SDQ; PTSD -C | Quantitative study; interview |
| 29 | Cluver et al., 2009 [38] | South Africa | 1050 children (HIV-affected) | 10 – 19 | Individual and social | CDI; CMAS; SDQ; PTSD-C; Social and Health | Quantitative study; interview |

| | | | | | | | |
|---|---|---|---|---|---|---|---|
| | | | | | | Assessment Peer Victimization Scale; Berger Stigma Scale for HIV-positive youth–Revised | |
| 30 | Nordtveit, 2010 [18] | Namibia and eSwatini (Swaziland) | 70 children 40 adults | 8 – 17 | Individual and social | School as a protective factor and HIV context. Unstructured, semi structured, and open-ended questions | Qualitative studies: 2 cases studies in Namibia and Swaziland (eSwatini) |
| 31 | Mueller et al., 2011 [28] | South Africa | 297 children (HIV-affected) | 8 – 18 | Individual and social | RSES; SEQ; CDI; SDQ; Snider scale for Stigma, social connection and experiencing violence; Social and Health Assessment Scale, psychosociodemo graphic characteristics | Quasi experimental project: art to increase self-esteem and self-efficacy |
| 32 | Okawa, 2011 [30] | Kenya | 398 children (orphaned by HIV/AIDS) pairs with caregivers | 10 – 18 | Individual and social | MSPSS; CES-DC; RSES | Cross-sectional study |
| 33 | Kagotho et al., 2012 [53] | Uganda | 297 caregivers | 18 – 87 | Individual, and social | Depression | Longitudinal study |
| 34 | Pufall et al., 2014 [65] | Zimbabwe | 4577 children (HIV-affected, HIV-positive) | 6 – 17 | Individual and social | CWI; monitoring and evaluation facility survey | Data from a cross-sectional household survey were linked to data on schools from a parallel monitoring and evaluation facility survey |

| | | | | | | | |
|---|---|---|---|---|---|---|---|
| 35 | Thurman et al., 2014 [40] | South Africa | 1487 children (HIV/AIDS-affected) 918 caregivers | 10 – 17 | Individual and social | CESDC; World Health Organization Quality of Life subscale | Longitudinal quasi-experimental design |
| 36 | Nicholson et al., 2015 [66] | Zambia | 390 children (111 HEU, 279 HUU) | 6 – 12 | Individual and social | General health, school report | Cross-sectional study |
| 37 | Sseswamala et al., 2016 [69] | Uganda | 346 children (orphaned by HIV/AIDS) | 12 – 16 | Individual and social | School primary leaving examination; child confidence in achieving educational plans; Beck hopelessness scale; Tennessee self-concept scale | Randomized experiment case control study |
| 38 | Scorza et al., 2017 [61] | Rwanda | 367 children (HIV-affected) | 10 – 17 | Individual and social | CD-RISC; protective and risk factors | Qualitative study |
| 39 | Sherr et al., 2017 [44] | Malawi and South Africa | 588 children (HIV-positive, HIV-affected, HUU) | 4 – 12* | Individual and social | Draw-a-person Test; Digit Span Test | Quantitative study |
| 40 | Sherr et al., 2017 [70] | Malawi and South Africa | 854 children (HIV-affected) | 5 – 15 * | Individual and social | CSI; Educational risk; grant receipt; number of grants; good parenting; Draw a Person test | Cash grant intervention |
| 41 | Phaladze et al., 2018 [67] | Botswana | 732 children (orphaned by HIV/AIDS) | 10 – 18 | Individual and social | School grade, loss and grief, living arrangement | Qualitative study; interview |
| 42 | Sharp et al., 2018 [43] | South Africa | 750 children (224 orphaned by HIV/AIDS, 276 orphaned by non-HIV/AIDS causes, 250 not orphaned) | 7 – 11 | Individual and social | SDQ; School Connectedness Scale | Cross-sectional study |

| | | | | | | | |
|---|---|---|---|---|---|---|---|
| 43 | Webster et al., 2018 [57] | Uganda | 168 children (HEU, HUU) 106 caregivers (HIV-positive, HIV-negative) | 6 – 18 | Individual and social | Distress Weinberger Adjustment Inventory; Children's Hopelessness Scale; Positive Outlook-Individual Protective Factors Index; Hare Area-specific self-esteem scale; PedsQLTM | Cross- sectional study |
| 44 | Katisi et al., 2019 [68] | Botswana | 650 children (orphaned by HIV/AIDS) | 11 – 17 | Individual and social | Child and Youth Resilience Measure-28; 5-point Inventory of Complicated Grief ; 3-point Future Aspirations/Peer Leader Survey | Quasi-experimental; pre-test and post-test |
| 45 | Small et al.,.2019 [32] | South Africa | 475 children (HIV-affected) | 9 – 13 | Individual and social | General health questionnaire; global indicator of well-being; CBCL; Neighborhood Social Control Scale; Neighborhood Social Cohesion Scale; Neighbourhood Disorganization Scale | Longitudinal study |
| 46 | Lachman et al., 2014 [54] | South Africa | 2477 children-caregivers dyads (HEU, HUU) | 10 – 17 >18 | Individual, family, and social | APQ; BNS 8-items about poverty; CESD, MOS-SSS; SDQ | Cross-sectional survey |
| 47 | Anabwani et al., | Botswana | 1238 children | 6 – 18 | Individual, family, | Questions about | Survey interview |

| | | | | | | | |
|---|---|---|---|---|---|---|---|
| | 2016 [63] | | (984 children HIV-positive, 254 HIV-affected) | | and social | school, activities, care, and stigma | |
| 48 | Hensels et al., 2016 [41] | Malawi and South Africa | 979 children (HIV-affected) | 4 – 13* | Individual, family, and social | CSI; P-C CTS; SDQ; ISPCAN; CDI; RSES; TSCC; Experience of Stigma, Discrimination and Social Exclusion Domain; PedsQL Draw-a-person Test; Digit Span Test; Ten Questions disability questionnaire | Longitudinal study |
| 49 | Ashry et al., 2017 [33] | Egypt | 65 children (33 HEU, 32 HUU) | 3 – 17* | Individual, family, and social | HRQOL; KINDL | Cross sectional comparative study |
| 50 | Slogrove et al., 2020 [7] | Global | HEU children | N/A | Individual, family, and social | N/A | Report |

**Table S1.** Summary of the 50 studies analysed in the present systematic review.
Acronyms used: Child Depression Inventory (CDI); Revised Children's Manifest Anxiety Scale (R-CMAS); Strengths and Difficulties Questionnaire (SDQ); Post-Traumatic Stress Disorder (PTSD); Child Behavior Checklist Youth Self-Report (CBCL YSR); Beck Hopeless Scale (BHS); Vineland Adaptive Behavior Scales (VABS); Child Behavior Checklist (CBCL); Revised-Children's Manifest Anxiety Scale (RCMAS); Bar-On Emotional Quotient Inventory: Youth Version (BOEQI YV); World Health Organization Disability Assessment Schedule for Children (WHODAS-Child); Centre for Epidemiologic Studies Depression Scale for Children (CESDC); Achenbach Youth Self Report (YSR); subscales of South African Child Assessment Schedule (SACAS); Psycho-Social Adjustment Scale-Adolescents (PAS-A); Reynolds Adolescent Depression Scale 2 (RADS-2); Behaviour Rating Inventory of Executive Function (BRIEF); Automatic Working Memory Assessment (AWMA); English Language Proficiency (ELP); Pediatric Quality of Life inventory (PedsQL ); Penn Computerized Neurocognitive Battery (PennCNB); Paediatric Symptom Checklist, (PSC); Brain Powered Games (BPG); Computerized Cognitive Rehabilitation Therapy (CCRT); Connor-Davidson Resilience Scale (CD-RISC); Multiple Indicator Cluster Survey-Round 4 (MICS4); Inventory of Socially Supportive Behaviors (ISSB); Irritability Questionnaire (IRQ); Youth Conduct Problems Scale-Rwanda Short Form (YCPS-RS); Rosenberg Self-Esteem Scale (RSE); Self-Efficacy Questionnaire for Children (SEQ-C); adapted Strauss Conflict Tactics

Scale, Parent-Child version (P-C CTS); Medical Outcomes Study Social Support Scale (MOS-SSS); Alabama Parenting Questionnaire (APQ); 12-item Short Form Survey (SF-12); Trauma Symptom Checklist for Children (TSCC); Social and Health Assessment (SAHA); Multidimensional Scale of Perceived Social Support (MSPSS); Center for Epidemiologic Studies Depression Scale for Children (CES-DC); Child Well-being Index (CWI); Connor Davidson Resilience Scale (CD-RISC); Mullen Scales of Early Learning (MSEL); Color Object Association Test (COAT); Early Childhood Vigilance Test (ECVT); Behavior Rating Inventory of Executive Function–Preschool version (BRIEF-P); Home Observation for the Measurement of the Environment (HOME); Hopkins Symptoms Checklist-25 (HSCL); Quality of Life (QOL); Basic Necessities Scale (BNS); Child Status Index (CSI); International Society for the Prevention of Child Abuse and Neglect (ISPCAN); Health-Related Quality of Life (HRQOL); Kinder Lebensqualität Fragebogen (KINDL).
